# Surface-Enhanced Raman Spectroscopic Investigation of PAHs at a Fe_3_O_4_@GO@Ag@PDA Composite Substrates

**DOI:** 10.3390/mi13081253

**Published:** 2022-08-04

**Authors:** Junyu Liu, Wencan Cui, Shihua Sang, Liang Guan, Kecheng Gu, Yinyin Wang, Jian Wang

**Affiliations:** 1 College of Material and Chemistry & Chemical Engineering, Chengdu University of Technology, Chengdu 610059, China; 2 Department of Petroleum, Oil and Lubricants Army Logistics Academy of PLA, Chongqing 401331, China; 3 Department of Basic Courses, Army Logistic Academy of PLA, Chongqing 401331, China

**Keywords:** surface-enhanced Raman spectroscopy, composite substrate, Ag, PAHs, enrichment detection

## Abstract

A method for surface-enhanced Raman spectroscopy (SERS) sensing of polycyclic aromatic hydrocarbons (PAHs) is reported. Fe_3_O_4_@PDA@Ag@GO is developed as the SERS substrate prepared by classical electrostatic attraction method based on the enrichment of organic compounds by graphene oxide (GO) and polydopamine (PDA) and the good separation and enrichment function of Fe_3_O_4_. The morphology and structure of the SERS substrate were represented by transmission electron microscopy (TEM), energy-dispersive spectroscopy (EDS), X-ray diffraction (XRD) and the UV–visible absorption spectrum (UV–vis spectra). The effect of different temperatures on SERS during synthesis was investigated, and it was found that the best effect was achieved when the synthesis temperature was 90 °C. The effect of each component of Fe_3_O_4_@PDA@Ag@GO nanocomposites on SERS was explored, and it was found that Ag NPs are of great significance to enhance the Raman signal based on the electromagnetic enhancement mechanism; apart from enriching the polycyclic aromatic hydrocarbons (PAHs) through π–π interaction, GO also generates strong chemical enhancement to the Raman signal, and PDA can prevent Ag from shedding and agglomeration. The existence of Fe_3_O_4_ is favored for the fast separation of substrate from the solutions, which greatly simplifies the detection procedure and facilitates the cycle use of the substrate. The experimental procedure is simplified, and the substrate is reused easily. Three kinds of PAHs (phenanthrene, pyrene and benzanthene) are employed as probe molecules to verify the performance of the composite SERS substrate. The results show that the limit of detection (LOD) of phenanthrene pyrene and benzanthene detected by Fe_3_O_4_@PDA@Ag@GO composite substrate are 10^−8^ g/L (5.6 × 10^−11^ mol/L), 10^−7^ g/L (4.9 × 10^−10^ mol/L) and 10^−7^ g/L (4.4 × 10^−10^ mol/L), respectively, which is much lower than that of ordinary Raman, and it is promising for its application in the enrichment detection of trace PAHs in the environment.

## 1. Introduction

Polycyclic aromatic hydrocarbons, which are widespread in the environment, are a kind of hydrocarbon containing two or more conjoined aromatic rings [1]. PAHs are mainly produced during the incomplete combustion of organic matter. Their carcinogenic, teratogenic and mutagenic effects are great threats to human health [2,3,4,5]. Nsibande et al. summarized the concentration of polycyclic aromatic hydrocarbons in various waters between 10^−6^–10^−11^ mol/L [6].

Therefore, the monitoring of the content and types of PAHs in the environment has attracted extensive attention. The detection methods for PAHs, such as gas chromatography, high-performance liquid chromatography (HPLC), gas chromatography-mass spectrometry (GC-MS), nuclear magnetic resonance (NMR) [7,8,9,10,11,12,13], etc., are accurate and used widely. Although the detection methods mentioned above have high sensitivity and stability, they usually demand expensive instrument and complex pre-treatment and enrichment processes, which are time-consuming. These shortcomings bring difficulties to the universal application of these methods. Therefore, a rapid and economical method with high-sensitivity and high-selectivity for PAH detection is necessary.

As an analytical technique with high sensitivity and strong feature recognition ability, surface-enhanced Raman spectroscopy (SERS) has been used widely in rapid detection [14,15,16,17,18]. According to the SERS enhancement mechanism, the target molecules must usually adsorb onto the surface of the SERS substrate by physical or chemical means or be in a short effective enhancement range. However, it is difficult to detect SERS substrates by conventional methods [16] because hydrocarbons such as PAHs do not have special functional groups that interact with metal substrates. Especially when the content of SERS is low, the difficulty of detection increases significantly. Therefore, surface functionalization of SERS substrate is necessary. At present, nanomaterials of noble metals (Au and Ag), transition metals and non-metallic graphene are mainly used to prepare substrates [19,20,21,22,23,24,25,26,27,28,29,30,31].

Currently, one of the most used methods mainly focuses on fixing PAH molecules on SERS substrate surfaces by self-assembly layer modification. For example, Harris et al. modified the metal substrate with the substitution of C18, and, with the help of portable Raman spectrometer, SERS detection of polycyclic aromatic hydrocarbon molecules pyrene and phenanthrene was carried out, with detection limits of 10^−8^ mol/L and 10^−7^ mol/L, respectively [32]. Jones et al. and Costa et al. assembled a layer of mercaptan molecules on Ag and Au membrane substrates by self-assembly. The minimum concentrations detected by pyrene are 7 × 10^−10^ mol/L [31,33]. The ability of SERS substrate to capture PAH molecules was improved. Through the improvement of nanoparticles, Jing et al. and Wang et al. prepared Fe_3_O_4_@Ag magnetic nanoparticles modified by thiol molecules and “bowl-shaped” Ag substrates modified by thiol, respectively, realizing the highly sensitive detection of PAHs molecules [34,35]. Wang et al. prepared an Ag-nanoparticle–graphene hybrid for the direct detection of PAHs. The synthesis process is relatively simple, and the raw materials are not expensive, while the limit of detection for pyrene, phenanthrene are as low as 0.73 ppb and 0.57 ppb, respectively [36].

Although this method has achieved SERS detection of PAHs, the organic molecules used for modification are usually difficult to synthesize, which limits the application of SERS.

Until now, the combination of Fe_3_O_4_, PDA, GO and Ag as a SERS substrate for the detection of PAHs has not been reported. PDA is a substance with rich functional groups, good hydrophilicity and excellent biocompatibility, which can cover many organic and inorganic materials such as magnetic nanomaterial Fe_3_O_4_. The existence of Fe_3_O_4_ can separate the substrate quickly and simplify the experimental steps. Graphene oxide (GO) is also known as functional graphene which has a two-dimensional spatial structure and is a carbon-based nanomaterial with abundant hydroxyl, epoxy, carboxyl and other oxygen-containing energy groups. It has high specific surface energy, good hydrophilic and mechanical properties and good adsorption of many chemicals. The accumulation and hydrophobic action of π–π between GO and PAHs can be used to enable the PAHs to show a strong enrichment ability. Ag and GO enhances the Raman signal in Fe_3_O_4_@PDA@Ag@GO substrate. PDA has good adhesion and reactivity. The composite nanoparticles have good magnetic response, which can quickly separate and enrich the molecules to be measured and shorten the pretreatment time.

In this study, Fe_3_O_4_@PDA@Ag@GO composite material was synthesized by assembly method to detect phenanthrene, pyrene and benzoanthracene in aqueous solution. The limit of detection for phenanthrene, pyrene and benzoanthracene are 10^−8^ g/L (5.6 × 10^−11^ mol/L), 10^−7^ g/L (4.9 × 10^−10^ mol/L) and 10^−7^ g/L (4.4 × 10^−10^ mol/L), respectively, which is much lower than that of ordinary Raman (Wang et al. prepared an Ag-nanoparticle/graphene hybrid for the direct detection of PAHs. The limit of detection for phenanthrene is 3.2 nM (~0.57 ppb) [24]). Our team synthesized Fe_3_O_4_@PDA@Au@GO substrate for the detection of phenanthrene. The limit of detection (LOD) of phenanthrene is 10^−^^7^ g/L. The raw materials for Fe_3_O_4_@PDA@Ag@GO substrates are much cheaper than those of Fe_3_O_4_@PDA@Au@GO.

## 2. Experimental

### 2.1. Materials and Apparatus

Materials and apparatus used in the experiment are shown in the following Table 1 and Table 2:

### 2.2. Material Synthesis

#### 2.2.1. Synthesis of Fe_3_O_4_ Nanoparticles

A total of 0.9 g of FeCl_3_·6H_2_O was dissolved in 28 mL of ethylene glycol (EG) by 15 min ultrasonic vibration to make FeCl_3_·6H_2_O completely dissolve. Then, 2.4 g of anhydrous sodium acetate and 0.2 g of polyethylene glycol (PEG) were added to the FeCl_3_ solution and stirred for 30 min. Ultrasound was performed for 15 min after stirring PEG for 30 min, for PEG was difficult to dissolve. Finally, the mixed solution was transferred to a 50 mL high-pressure reactor and continuously heated to 200 °C in an electric blast-drying oven for 8 h. Then, the blast-drying oven was turned off and the solution was cooled at room temperature naturally. The Fe_3_O_4_ solution was poured into a beaker to separate the Fe_3_O_4_ nanoparticles by a magnet and the supernatant was removed. Then, Fe_3_O_4_ nanoparticles were washed with ultrapure water and anhydrous ethanol, alternately, 3 times, and were dried at 60 °C for 6 h in an electric blast-drying oven [1,18,19].

#### 2.2.2. Synthesis of Fe_3_O_4_@PDA

First, 2 mg dopamine was added to 6 mL Tris-HCl buffer solution (pH = 8.4) and ultrasound to obtain a uniform PDA@Tris-HCl dispersion. Next, 40 mg Fe_3_O_4_ nanoparticles were added to the solution under constant ultrasound until they were completely dissolved in the PDA/Tris-HCl solution, and, then, after magnetic stirring for 3 h, Fe_3_O_4_@PDA products were separated from the solution by an external magnetic field. Lastly, the as-obtained products were washed with ultrapure water three times and dried at 50 °C for 12 h to yield Fe_3_O_4_@PDA [20].

#### 2.2.3. Synthesis of Fe_3_O_4_@PDA@Ag

First, 10 mg Fe_3_O_4_@PDA was added to 20 mL ultrapure water to obtain a uniform dispersion under ultrasound. Next, 100 mL 0.001 mol/L AgNO_3_ solution was poured gently into the solution mentioned above with constant stirring and heating [19]. When the solution temperature reached 90 °C, 0.0258 g sodium citrate solution was added, and the reaction took 1.5 h. Fe_3_O_4_@PDA@Ag products were separated from the solution by an external magnetic field. Eventually, the as-obtained products were washed with ultrapure water 3 times and dried at 50 °C for 12 h to yield Fe_3_O_4_@PDA@Ag [16].

#### 2.2.4. Synthesis of Fe_3_O_4_@PDA@Ag@GO

First, 10 mg of Fe_3_O_4_@PDA@Ag was dissolved in water, 0.5 mL 0.5 mol/L of GO dispersion was added, and ultrapure water was added to make the volume of solution 12 mL with magnetic stirring for 3 h. Next, the as-obtained products were washed with ultrapure water 3 times [20], and Fe_3_O_4_@PDA@Ag@GO products were separated from the solution by an external magnetic field. At last, ultrapure water was added to make the volume of solution 10 mL [16].

## 3. Results and Discussion

### 3.1. Characterization

#### 3.1.1. UV-Visible Characterization

**Figure 1:**
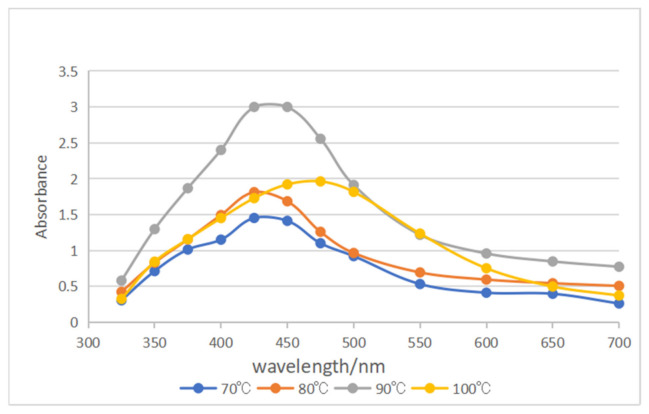
UV absorption spectra of silver dissolved at different temperatures.

The UV-vis spectra in Figure 1 illustrate the maximum absorption of Ag NPs synthesized by various reaction temperatures, which are located at the range of 300~700 nm to study the effect of reaction temperature on the preparation of silver sol. The reaction temperatures were 70 °C, 80 °C, 90 °C and 100 °C, respectively.

The prepared AgNO_3_ solution (0.001 mol/L, 100 mL) were added to four beakers, respectively, and 0.0258 g sodium citrate were added to each of the four beakers and were heated, respectively, to 70 °C, 80 °C, 90 °C and 100 °C for 1.5 h.

It was observed that the color of the solution heated to 70 °C was lighter than others, and precipitation occurred in the solution that was heated to 100 °C. It is shown in Figure 1 that the strongest absorption peaks of the four beakers of silver sol were located between 400~500 nm, and the strongest absorption peak for each of the silver sol was located at 425 nm when the solution was heated to 70 °C, 80 °C and 90 °C. When the silver sol was heated to 70 °C, the concentration was low for the reaction was incomplete. When the temperature was as high as 100 °C, the strongest absorption peak moved to right and the concentration was also low for the aggregate sank to the bottom. The preparation of Ag NPs followed the procedure reported in the literature [37,38]. Therefore, the most suitable heating temperature for synthesizing Fe_3_O_4_@PDA@Ag is 90 °C.

#### 3.1.2. TEM Image of Fe_3_O_4_@PDA@Ag@GO

The size, morphology and surface composition of Fe_3_O_4_@PDA@Ag@GO were characterized by TEM (FEI Talos F200×, FEI Company, Hillsborough, OR, USA) [16].

**Figure 2:**
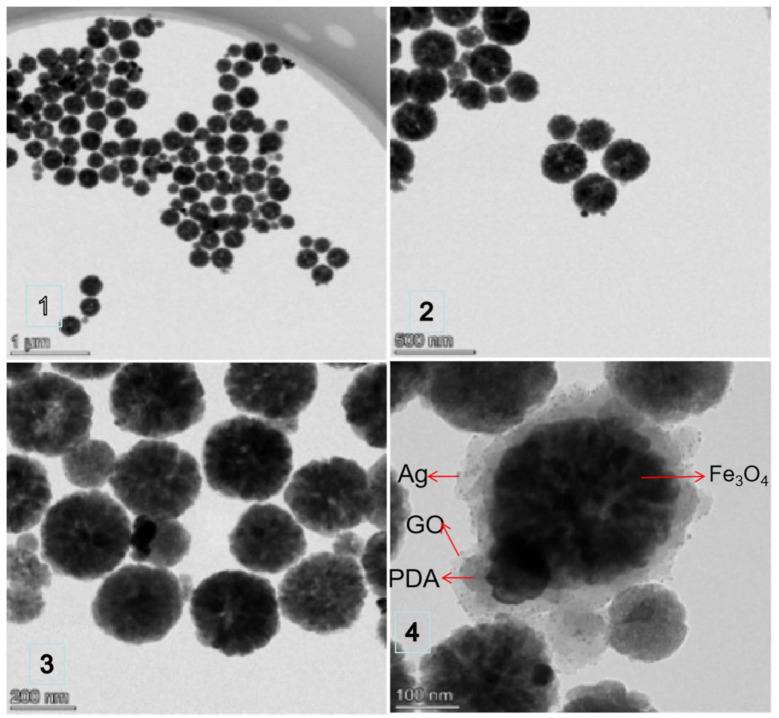
TEM images Fe_3_O_4_@PDA@Ag@GO of different magnifications. (**1**) TEM image zoomed to 1 μm; (**2**) TEM image zoomed to 500 nm; (**3**) TEM image zoomed to 200 nm; (**4**) TEM image zoomed to 100 nm.

**Figure 3:**
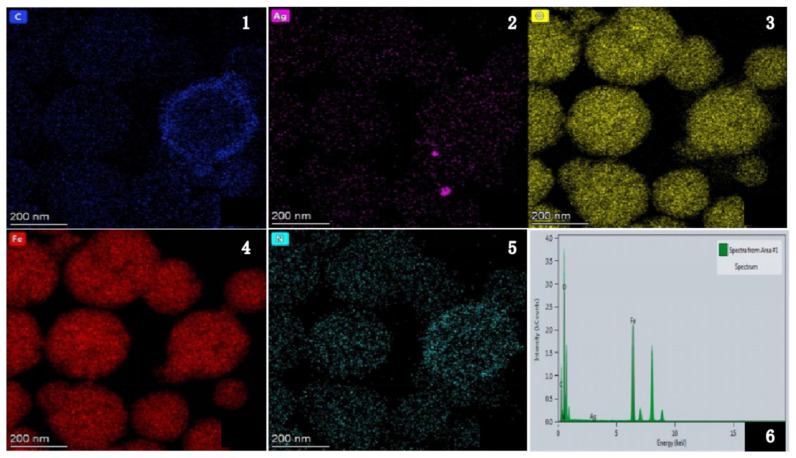
TEM mapping and EDS of Fe_3_O_4_@PDA@Ag@GO nanocomposites. (**1**) The elemental mappings of C; (**2**) the elemental mappings of Ag; (**3**) the elemental mappings of O; (**4**) the elemental mappings of Fe; (**5**) the elemental mappings of N; (**6**) EDS of Fe_3_O_4_@PDA/Ag/GO.

As is shown in Figure 2 and Figure 3, most nanoparticles are spherical, with magnifications of 1 μm, 500 nm, 200 nm and 100 nm, respectively. AgNPs are evenly distributed on the GO surface without large aggregate generation, which indicates that Ag NPs can be adsorbed effectively on the GO surface and GO plays a good role in dispersing Ag NPs.

When zoomed to 100 nm, as is shown in the following pictures, Fe_3_O_4_ is clustered in the innermost layer. The interlayer between globular Fe_3_O_4_ and Ag nanoparticles is PDA, the black dots are Ag nanoparticles and the outermost layer is GO.

The peaks of Ag, Fe, C and O can be obtained by EDS analysis of Fe_3_O_4_@PDA@Ag@GO nanocomposite in (6) in the Figure 3, which proves that there are Ag, Fe, C and O in the substrate and Fe_3_O4@PDA@Ag@GO was successfully synthesized [21].

#### 3.1.3. XRD Characterization

The crystal phase was characterized by D8-Advance X-ray diffraction (XRD, Bruker, Germany, Copper Kα radiation). The range of the scanning angle was (5~85)° and the scanning rate was 8°/min [20].

**Figure 4:**
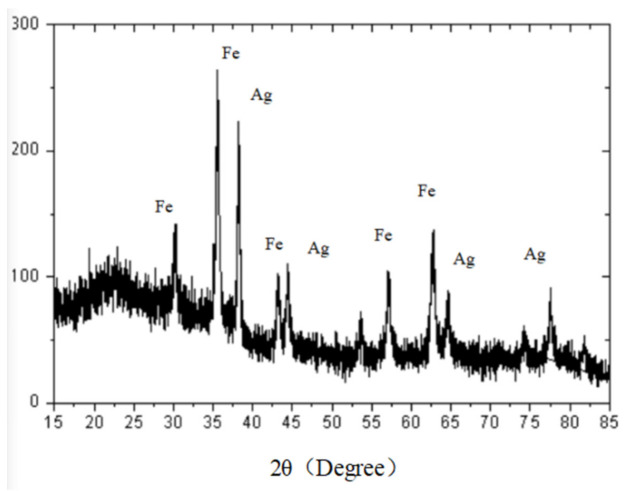
XRD of Fe_3_O_4_@PDA@Ag@GO.

Figure 4 shows the XRD patterns of Fe_3_O_4_@PDA@Ag@GO. The corresponding peaks of Fe_3_O_4_ crystal occurred at 30°, 35°, 43°, 57° and 62°. The peaks corresponding to silver crystals were at 38°, 44°, 64° and 77°, which were consistent with the peaks in the literature. According to the XRD analysis above, there were Fe_3_O_4_ nanoparticles and Ag nanoparticles in SERS substrate [22].

### 3.2. Detection of PAHs with SERS Substrate

#### 3.2.1. Sample Preparation

First, 0.1 g of PAHs were added to a moderate amount of alcohol under ultrasound for 30 min and then magnetically stirred for 2 h. Next, the as-prepared solution was poured into a 100 mL volumetric bottle with constant volume to prepare a PAHs methanol solution with a concentration of 1 g/L. Finally, PAHs solutions of different concentrations were prepared by diluting 1 g/L of PAH methanol solution with methanol and ultrapure water [16].

#### 3.2.2. Method for Detection

Raman spectroscopy was performed on a Metage Opal 2800 Raman spectrometer with an excitation wavelength of 785 nm. First, the portable spectrometer was turned on and preheated for 30 min in order to check whether the instrument and laser work properly. Next, the PAHs solution and the prepared Fe_3_O_4_@PDA@Ag@GO substrate were added to a brown sample bottle in proportion. The integral time was set for 15~30 s, and the laser intensity was set at 90. Then, the average value of each sample was shown after the value of the sample was recorded 4 times. Finally, the Raman spectrum was obtained when the background was subtracted, and the dark current was deducted.

#### 3.2.3. The Detection of Phenanthrene

**Figure 5:**
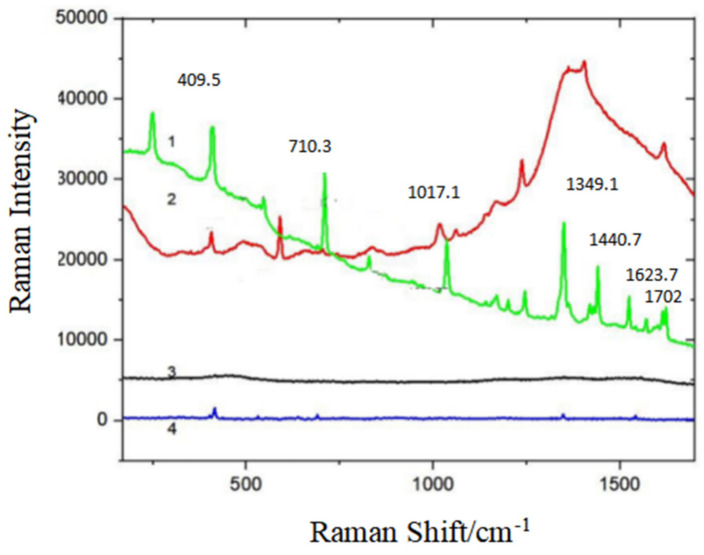
SERS spectra of phenanthrene (**1**) phenanthrene solid; (**2**) the mixture of 10^−2^ g/L phenanthrene and SERS substrate solution; (**3**) Fe_3_O_4_@PDA@Ag@GO solution; (**4**) 10^−2^ g/L phenanthrene solution.

Figure 5 illustrates the influences of the synthesis of Fe_3_O_4_@PDA@Ag@GO material on the Raman enhancement of PAHs solution. Phenanthrene solid, 10^−2^ g/L phenanthrene solution, the mixture of 10^−2^ g/L phenanthrene solution and SERS substrate and Fe_3_O_4_@PDA@Ag@GO solution were detected. The detection results are displayed in Figure 5.

PAHs can be differentiated based upon their characteristic SERS peaks [39]. The 10^−2^ g/L phenanthrene solution ((4) in the Figure 5) has a characteristic peak with a low intensity, while the characteristic peak of Fe_3_O_4_@PDA@Ag@GO substrate ((3) in the Figure 5) is negligible. When 10^−2^ g/L phenanthrene solution is mixed with Fe_3_O_4_@PDA@Ag@GO substrate, an obvious Raman characteristic peak ((2) in the Figure 5) appears, which is located at 402.7 cm^−1^, 1017.12 cm^−1^, 1238.64 cm^−1^, 1405.63 cm^−1^ and 1614.98 cm^−1^ that corresponds to the peak of phenethene solid ((1) in the Figure 5). The results show that Fe_3_O_4_@PDA@Ag@GO substrate enhances the adsorption capacity of 10^−2^ g/L phenanthrene standard solution, and the Raman effect on the surface phenanthrene is obviously enhanced.

1. Effects of each component in Fe_3_O_4_@PDA@Ag@GO on SERS detection performance

**Figure 6:**
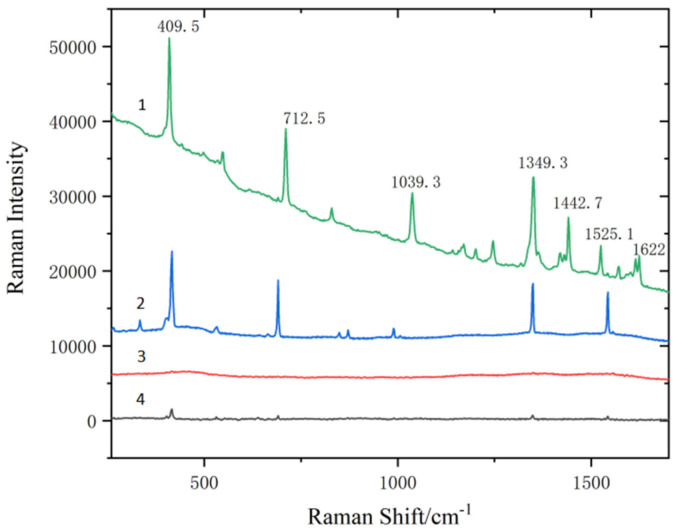
SERS signal of phenanthrene captured on Fe_3_O_4_@PDA@Ag@GO, Fe_3_O_4_@PDA@Ag, Fe_3_O_4_@PDA and Fe_3_O_4_, respectively (1) Fe_3_O_4_@PDA@Ag@GO substrate; (2) Fe_3_O_4_@PDA@Ag substrate; (3) Fe_3_O_4_@PDA substrate; (4) Fe_3_O_4_ substrate.

It is illustrated by (3) in the Figure 6 and (4) in the Figure 6 that the characteristic peak is negligible. Fe_3_O_4_ and Fe_3_O_4_@PDA do not enhance the SERS activity of the phenanthrene solution of 10^−2^ g/L. This may result from the fact that PDA covered Fe_3_O_4_, thus the SERS activity of Fe_3_O_4_ not being shown. It is illustrated by (1) in the Figure 6 and (2) in the Figure 6 that Fe_3_O_4_@PDA@Ag@GO has a stronger Raman signal than Fe_3_O_4_@PDA@Ag. Fe_3_O_4_@PDA@Ag@GO substrate has a combined enhancement of the electromagnetic enhancement of Ag and the chemical enhancement of GO. The adsorption capacity of the phenanthrene standard solution of 10^−2^ g/L is increased and the sensitivity is improved.

2. The influence of the mixing ratio of SERS substrate and phenanthrene solution on SERS detection performance

In the experiment, the ratio of the mixture of the substrate and the solution also affected the value of Raman spectral peak. The volume ratio of the substrate and the solution was studied to find the optimal ratio. The mixed solutions of the different volume ratio of 2:1, 1:1, 1:2 and 1:4 were used to study the influence of the volume ratio of the mixture of SERS substrate with phenanthrene solution on SERS detection performance.

**Figure 7:**
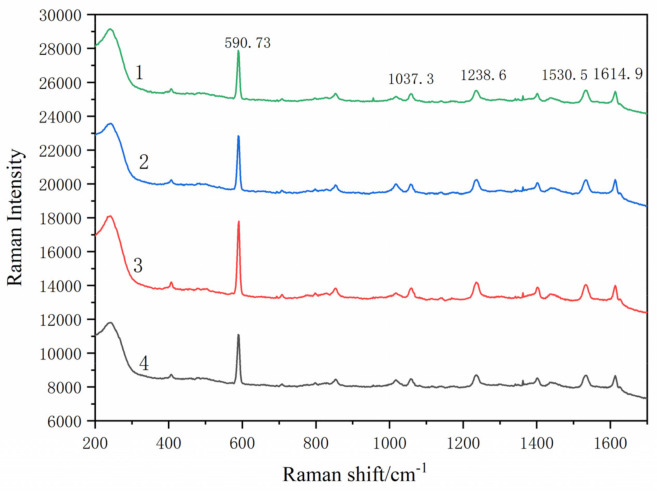
SERS spectra of different mixing volume ratios of Fe_3_O_4_@PDA@Ag@GO substrate and phenanthrene solution (1) 2:1;(2) 1:1; (3) 1:2; (4) 1:4.

As shown in Figure 7, Raman spectral peaks of the mixed solution of the substrate and the phenanthrene solution change with their different volume ratios. Raman characteristic peaks are located at 590.7 cm^−1^,1037.3 cm^−1^, 1238.6 cm^−1^,1530.5 cm^−1^ and 1614.9 cm^−1^.The Raman displacement of the Raman spectral peak of the solution has shifted, possibly due to fluorescence interference. The peak heights of the spectrum from 1 to 4 are 2570.4, 3157.2, 4293.7 and 2904.6, respectively. Therefore, the volume ratio corresponding to spectrum 3 is chosen for the optimal ratio to be ready for the next step of the experiment.

Raman spectral intensity is the strongest when the volume ratio of substrate to standard solution is 1:2. The intensity of Raman spectrum peak reduces as the amount of substrate decreases. The number of molecules in hot spots on SERS substrate increases with the increase in the amount of the substrate. Therefore, the Raman intensity is intensified significantly. However, when the concentration of the substrate increases to a certain value and the enriched phenanthrene solution reaches the limit, the substrate interferes with the Raman signal, thus decreasing the spectral intensity.

3. Fe_3_O_4_@PDA@Ag@GO detection limit for phenanthrene

**Figure 8:**
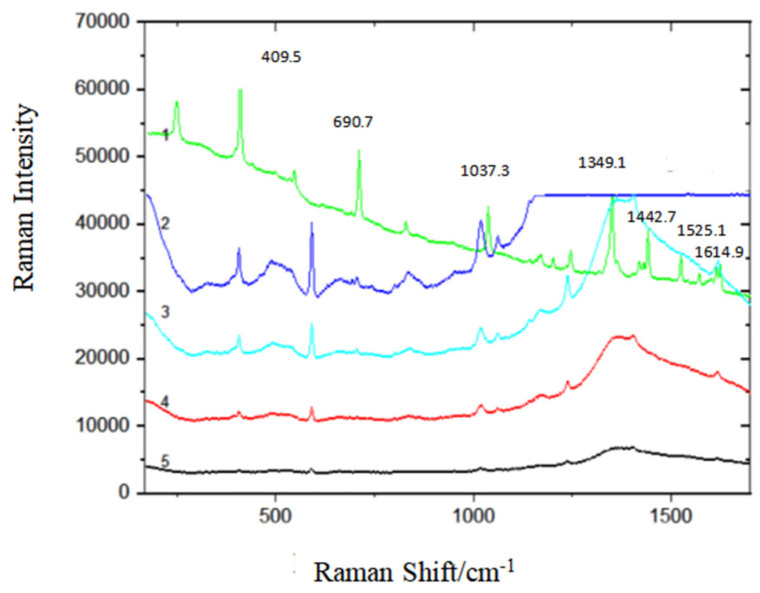
The detection limit of phenanthrene captured on Fe_3_O_4_@PDA@Ag@GO nanocomposites SERS substrate (1) phenanthrene solid; (2) 10^−5^ g/L phenanthrene SERS solution; (3) 10^−6^ g/L phenanthrene SERS solution; (4) 10^−7^ g/L phenanthrene SERS solution; (5) 10^−8^ g/L phenanthrene SERS solution.

In order to explore the influence of the synthesis of Fe_3_O_4_@PDA@Ag@GO material on the Raman enhancement of phenanthrene solution and to detect the detection limit of phenanthrene, the phenanthrene solution at different concentrations from 10^−5^ g/L to 10^−8^ g/L is mixed with the substrate of Fe_3_O_4_@PDA@Ag@GO and the Raman spectra is captured as shown in Figure 8.

As shown in Figure 8, the intensity of characteristic peak of the mixture of the phenanthrene solution (10^−5^~10^−8^ g/L) and the substrate decreases as the concentration of the solution is decreased. When the concentration of the phenanthrene solution reaches 10^−8^ g/L, a slight characteristic peak can still be seen, thus demonstrating that the detection limit of Fe_3_O_4_@PDA@Ag@GO for phenanthrene solution can reach 10^−8^ g/L.

**Figure 9:**
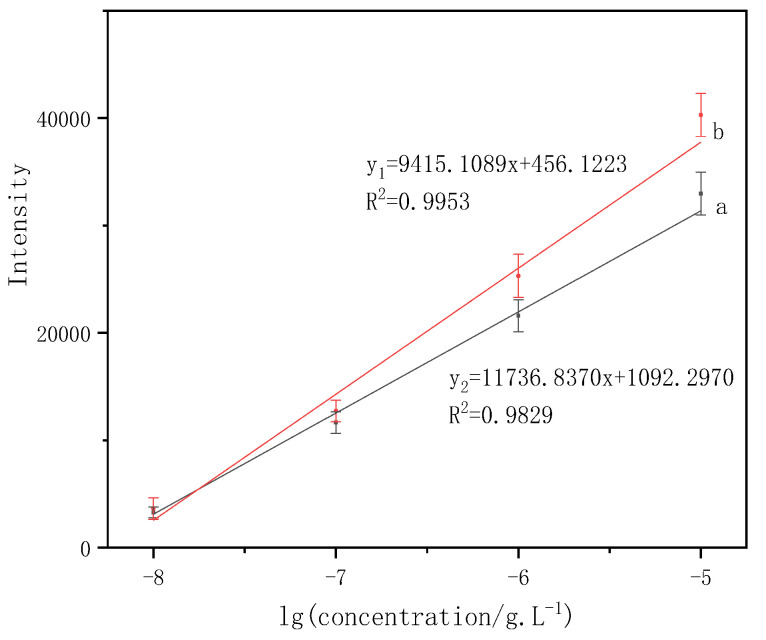
Linear relationships between the phenanthrene characteristic intensity and the logarithm of phenanthrene concentration. (a) 590 cm^−1^; (b) 411 cm^−1^.

In order to study the linear relationship between the intensities of characteristic peaks and the logarithm of phenanthrene concentration, two characteristic peaks of phenanthrene, 411 cm^−1^ and 590 cm^−1^, were analyzed which was shown in Figure 9.

The SERS peak intensities versus the logarithm of phenanthrene concentration had a good linear relationship from 10^−8^ to 10^−5^ g/L (Figure 9). The calibration equation can be described as (the characteristic absorption peaks located at 411 cm^−1^) y_1_ = 9415.1089x + 456.1223. The linear regression equation (the characteristic absorption peaks located at 590 cm^−1^) is y_2_ =11,736.837x + 1092.2970. The error bars indicate that the standard is derived from a total of 10 measurements. The present results reveal the potential for this method to be a rapid analysis and detection of phenanthrene at low concentrations.

#### 3.2.4. Limit of Detection for Pyrene

**Figure 10:**
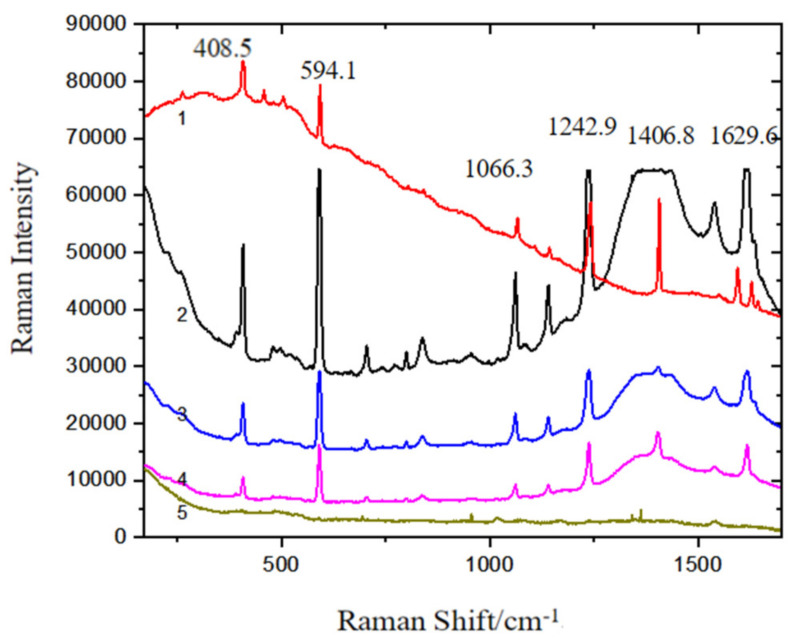
The detection limit of Pyrene captured on Fe_3_O_4_@PDA@Ag@GO nanocomposites SERS substrate (1) Pyrene solid; (2) 10^−5^ g/L Pyrene SERS solution; (3) 10^−6^ g/L Pyrene SERS solution; (4) 10^−7^ g/L Pyrene SERS solution; (5) 10^−8^ g/L Pyrene SERS solution.

In order to explore the influence of the synthesis of Fe_3_O_4_@PDA@Ag@GO material on the Raman enhancement of pyrene standard solution and to detect the detection limit of pyrene, the pyrene solution at different concentrations from 10^−5^ g/L to 10^−8^ g/L is mixed with the substrate of Fe_3_O_4_@PDA@Ag@GO and the Raman spectra is captured as shown in Figure 10.

As shown in Figure 10, the intensity of the characteristic peak of the mixture blending the pyrene standard solution (10^−5^~10^−8^ g/L) with the substrate decreases with the decrease of the concentration of the solution. When the concentration of the pyrene standard solution reaches 10^−7^ g/L, the spectral signal is apparent, and the characteristic peak is clearly visible. However, when the concentration reaches 10^−8^ g/L, the spectral signal disappears and almost becomes a straight line. Therefore, the detection limit of Fe_3_O_4_@PDA@Ag@GO for pyrene solution is 10^−7^ g/L.

**Figure 11:**
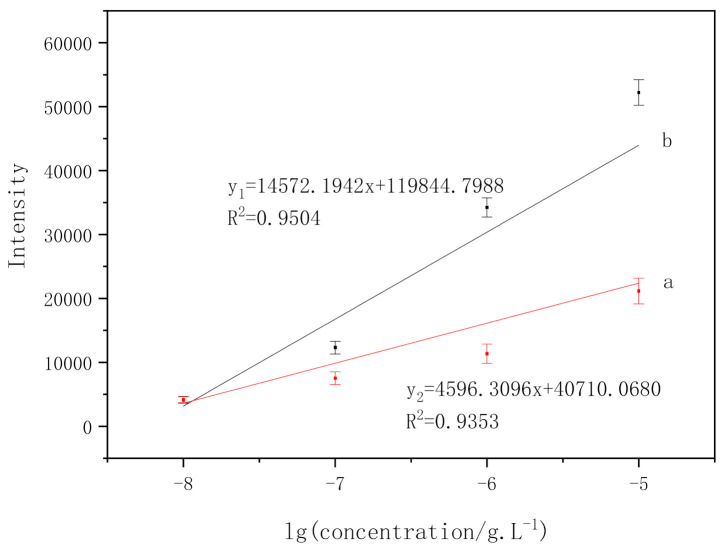
Linear relationships between the pyrene characteristic intensity and the logarithm of pyrene concentration. (a) 1016 cm^−1^; (b) 590 cm^−1^.

In order to study the linear relationship between the intensities of characteristic peaks and the logarithm of pyrene concentration, two characteristic peaks of pyrene, 590 cm^−1^ and 1016 cm^−1^, were analyzed which was shown in Figure 11.

**Figure 12:**
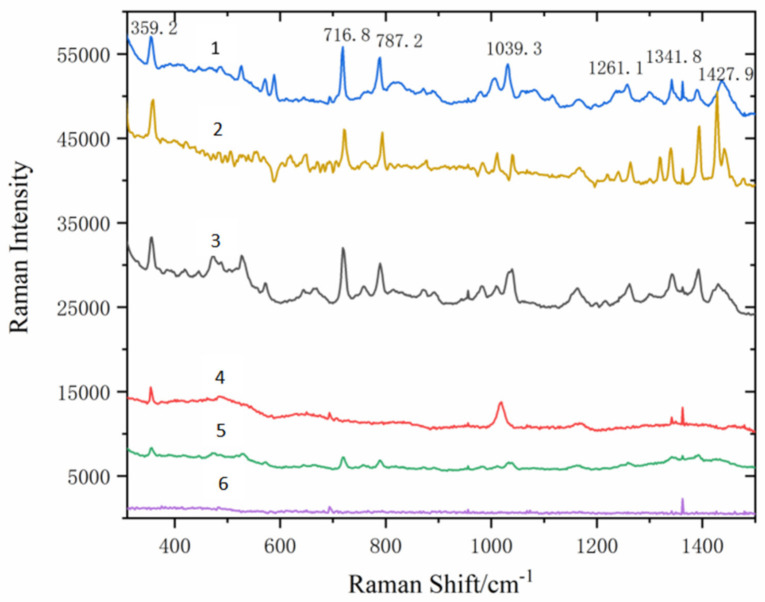
The detection limit of benzanthene captured on Fe_3_O_4_@PDA@Ag@GO nanocomposites SERS substrate (1) 10^−4^ g/L benzanthene standard solution; (2) benzanthene solid; (3) 10^−5^ g/L benzanthene standard solution; (4) 10^−6^ g/L benzanthene standard solution; (5) 10^−7^ g/L benzanthene standard solution; (6) 10^−8^ g/L benzanthene standard solution.

The SERS peak intensities versus the logarithm of pyrene concentration had a good linear relationship from 10^−8^ g/L to 10^−5^ g/L (Figure 12). The calibration equation can be described as (the characteristic absorption peaks located at 590 cm^−1^) y_1_ = 14,572.1942x + 119,844.7988. The linear regression equation (the characteristic absorption peak is located at 1016 cm^−1^) is y_2_ = 4596.3096x + 40,710.068. The error bars indicate that the standard is derived from a total of 10 measurements. The present results reveal the potential of this method to do rapid analysis and detection of pyrene at low concentrations.

#### 3.2.5. Detection Limit of Benzanthracene

In order to explore the influence of the synthesis of Fe_3_O_4_@PDA@Ag@GO material on the Raman enhancement of benzanthracene solution and to detect the detection limit of benzanthracene, the benzanthracene solution at different concentrations from 10^−5^ g/L to 10^−8^ g/L is mixed with the substrate of Fe_3_O_4_@PDA@Ag@GO and the Raman spectra is captured as shown in Figure 12.

As shown in Figure 12, the intensity of the characteristic peak of the mixture blending the benzanthracene solution (10^−5^~10^−8^ g/L) with the substrate decreases with the decrease of the concentration of the solution. When the concentration of the benzanthracene solution reaches 10^−7^ g/L, the spectral signal almost disappears, but a weak characteristic peak can still be seen. However, when the concentration reaches 10^−8^ g/L, the spectral signal disappears and almost becomes a straight line. Therefore, the detection limit of Fe_3_O_4_@PDA@Ag@GO for benzanthracene solution is 10^−7^ g/L.

**Figure 13:**
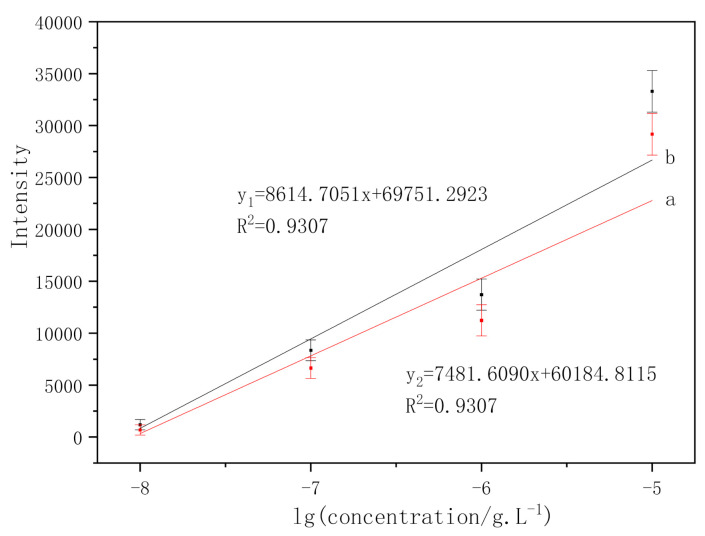
Linear relationships between the benzoanthracene characteristic intensity and the logarithm of benzoanthracene concentration. (a) 1033 cm^−1^; (b) 356 cm^−1^.

In order to study the linear relationship between the intensities of characteristic peaks and the logarithm of benzanthracene concentration, two characteristic peaks of benzanthracene located at 356 cm^−1^ and 1033 cm^−1^ were analyzed as shown in Figure 13.

The linear relationships between the SERS intensity and the logarithm of benzanthracene concentrations (from 10^−8^ g/L to 10^−5^ g/L) are detected in Figure 13. The intensity of the characteristic peak decreases with the decrease of concentration. The fitting linear relationship in line a (the characteristic absorption peaks located at 1033 cm^−1^) of Figure 13 is y_2_ = 7481.609x + 60,184.8115 while that in line b (the characteristic absorption peaks located at 356 cm^−1^) of Figure 13 is y_1_ = 8614.7051x + 69,751.2923. The x is the logarithm of solution concentration and the y is the relative intensity.

## 4. Conclusions

Fe_3_O_4_@PDA@Ag@GO substrate with high SERS activity was prepared and its morphology and structure were characterized in the experiment. By investigating the effects of different heating temperatures on the preparation of silver sol, it is discovered that the concentration of AgNPs is the highest when heated to 90 °C and the SERS signal intensity of surface polycyclic aromatic hydrocarbons (PAHs) is the highest. The study on the influence of different components of composite substrate on SERS also observes that Ag and GO enhanced the Raman signal in the Fe_3_O_4_@PDA@Ag@GO substrate [16]; the detection limit of phenanthrene is 10^−8^ g/L, that of pyrene 10^−7^ g/L and that of benzoanthracene 10^−7^ g/L, when Fe_3_O_4_@PDA@Ag@GO is used as SERS substrate. The composite nanoparticles have good magnetic response, can separate quickly and enrich the molecules to be measured and shorten the pretreatment time [16]. Therefore, Fe_3_O_4_@PDA@Ag@GO can be used as SERS substrate for the enrichment and detection of trace PAHs in the environment [23].

## Figures and Tables

**Table 1:** Reagents.

Reagent	Grade	Manufacturer
Ethylene glycol	analytical purity	Chengdu Colon Chemicals Co., Ltd. (Chengdu, China)
Methanol	analytical purity	Chengdu Colon Chemicals Co., Ltd. (Chengdu, China)
Anhydrous ethanol	analytical purity	Chengdu Colon Chemicals Co., Ltd. (Chengdu, China)
Polyethylene glycol	analytical purity	Beijing Chemical Reagent Co., Ltd. (Beijing, China)
Ferric chloride hexahydrate	analytical purity	Beijing Chemical Reagent Co., Ltd. (Beijing, China)
Silver nitrate	analytical purity	Beijing Chemical Reagent Co., Ltd. (Beijing, China)
Sodium citrate	analytical purity	Beijing Chemical Reagent Co., Ltd. (Beijing, China)
Phenanthrene	analytical purity	McLean Biochemical Technology Co., Ltd. (Shanghai, China)
Pyrene	analytical purity	McLean Biochemical Technology Co., Ltd. (Shanghai, China)
Benzanthracene	analytical purity	McLean Biochemical Technology Co., Ltd. (Shanghai, China)
Graphene oxide dispersion	0.5 mg/mL	Beijing Chemical Reagent Co., Ltd. (Beijing, China)
Hydrochloric acid-dopamine	analytical purity	McLean Biochemical Technology Co., Ltd. (Shanghai, China)
Anhydrous sodium acetate	analytical purity	Beijing Chemical Reagent Co., Ltd. (Beijing, China)

**Table 2:** Laboratory apparatus.

Apparatus Names	Manufacturers	Model Number
Electronic balances	METTLER TOLEDO, Zurich, Switzerland.	XP5003S
Thermostatic magnetic agitator	Jintan Fuhua Electric Appliance Co., Ltd.	RCT B S25
TG16-WS high speed centrifuge	Hunan Xiangyi Laboratory Instrument Development Co., Ltd.	TG16-WS
SB-5200DT Ultrasonic cleaner	Ningbo Xinzhi Biotechnology Co., Ltd.	SB-5200DT
Metage Opal 2800 spectrometer	Metage Co., London, England	Metage Opal 2800
50mL Stainless steel reactor	Chengdu Cologne Chemicals Co., Ltd.	PDFE
DZF vacuum oven	Beijing Yongguangming Medical Instrument Co., Ltd.	DZF
Electric blower drying box	Chongqing Star Test Equipment Co., Ltd.	CS101-2EB
Spectrophotometer	Shanghai Meipuda Instrument Co., Ltd.	UV-1600PC

## Data Availability

The data presented in this study are available on request from the corresponding author.
